# Enhancing wind direction prediction of South Africa wind energy hotspots with Bayesian mixture modeling

**DOI:** 10.1038/s41598-022-14383-8

**Published:** 2022-07-06

**Authors:** Najmeh Nakhaei Rad, Andriette Bekker, Mohammad Arashi

**Affiliations:** 1 Department of Statistics, University of Pretoria, Pretoria, 0002 South Africa; 2 DSI-NRF Centre of Excellence in Mathematical and Statistical Sciences (CoE-MaSS), Pretoria, South Africa; 3 Department of Statistics, Faculty of Mathematical Sciences, Ferdowsi University of Mashhad, Mashhad, Iran

**Keywords:** Climate sciences, Mathematics and computing

## Abstract

Wind energy production depends not only on wind speed but also on wind direction. Thus, predicting and estimating the wind direction for sites accurately will enhance measuring the wind energy potential. The uncertain nature of wind direction can be presented through probability distributions and Bayesian analysis can improve the modeling of the wind direction using the contribution of the prior knowledge to update the empirical shreds of evidence. This must align with the nature of the empirical evidence as to whether the data are skew or multimodal or not. So far mixtures of von Mises within the directional statistics domain, are used for modeling wind direction to capture the multimodality nature present in the data. In this paper, due to the skewed and multimodal patterns of wind direction on different sites of the locations understudy, a mixture of multimodal skewed von Mises is proposed for wind direction. Furthermore, a Bayesian analysis is presented to take into account the uncertainty inherent in the proposed wind direction model. A simulation study is conducted to evaluate the performance of the proposed Bayesian model. This proposed model is fitted to datasets of wind direction of Marion island and two wind farms in South Africa and show the superiority of the approach. The posterior predictive distribution is applied to forecast the wind direction on a wind farm. It is concluded that the proposed model offers an accurate prediction by means of credible intervals. The mean wind direction of Marion island in 2017 obtained from 1079 observations was 5.0242 (in radian) while using our proposed method the predicted mean wind direction and its corresponding $95\%$ credible interval based on 100 generated samples from the posterior predictive distribution are obtained 5.0171 and (4.7442, 5.2900). Therefore, our results open a new approach for accurate prediction of wind direction implementing a Bayesian approach via mixture of skew circular distributions.

## Introduction

The future of the energy industry lies in clean power that minimizes or entirely removes pollutants from the process of power generation. The perfect clean energy mix occurs where green energy, derived from natural sources, meets renewable energy from sources that are constantly being replenished. Wind energy is one of the most important sustainable forms of this ideal clean energy and one of the fastest-growing energy sources. A sophisticated knowledge, based on statistical analysis, of wind characteristics is crucial for the future harnessing of this important renewable energy resource. Wind power is developing as a renewable energy source in a number of countries and it will be increasingly important to find an effective and predictable way of integrating this intermittent but environmentally friendly power source into the existing electrical grid system.

In South Africa, there is an increasing transition towards an environmentally sustainable, climate-change resilient, low-carbon economy. In October 2020, the South African Wind Energy Association (SAWEA) reported that wind technology has already attracted R209.7 billion in investment for the development of projects in South Africa. In fact wind power comprises a larger share of the planned renewable energy investments to date. It is estimated by 2030 that $22.7\%$ of the required electricity in South Africa, namely 17742 MW, will be generated from wind energy. In terms of job creation, the 22 wind Independent Power Producers (IPPs) that have successfully reached commercial operations to date, have created 2723 jobs for South African citizens.

Wind as an energy source is only practical in areas that have strong and steady winds. South Africa’s climatology allows for significant wind energy production especially along the coastal areas of the Eastern and Western Capes. The first large-scale wind farm in South Africa became operational in 2014 and based on the SAWEA report, there are 33 wind farms: 22 fully operational and 11 in construction. In this paper, we will study the wind direction of two operational wind farms in South Africa: (1) Jeffreys Bay (Humansdorp), located in the Eastern Cape; (2) Noupoort located in the Northern Cape. In addition, we will investigate the wind direction data from Marion Island, part of the Western Cape Province which possesses excellent potential for wind studies.

Unlike conventional energy resources that are available at any time, wind speed and wind direction need to be forecasted in advance in order to estimate production and plan its contribution to a nation’s grid system. As the use of wind power increases, accurate forecasts are essential to maximize output from the wind farms. This includes the most important decision of all, the location of a wind farm and the placement of its turbines^1^.

The location of an industrial-scale wind farm, defined as a cluster of wind turbines used to produce electricity, is of paramount importance. Measuring the farm-specific wind characteristics including mean wind speed, wind speed distribution (diurnal, seasonal, annual patterns), distribution of wind direction, short-term fluctuations, long-term fluctuations and wind shear profile are essential for determining the location of farm and turbines. This can strongly influence the performance of the wind turbines and thus the power generated by the wind farms^2^. Moreover, interactions among multiple turbines change the power generation efficiency of turbines. Specifically, the wakes from upwind turbines can greatly affect the power production of downstream turbines, and this effect depends strongly on the wind direction^3^. Generally, downstream turbines produce less power compared to upwind turbines, but changes in wind direction can cause heterogeneity in the power curve of each turbine such that some upstream turbines can become downstream turbines^4^. Porté-Agel et al.^5^ presented a study about the effects of wind direction on turbine wakes and power losses at a large wind farm. Castellani et al.^6^ showed how the alignment of wind turbines to wind direction affects efficiency (see also Kazacoks et al.^7^ and Gomez and Lundquist^8^).

Predicting wind speed and wind direction are crucial to choose the location of wind farm and the placement of its turbines and also to estimate wind power production. To the best of the authors’ knowledge none of the existing literature follows a directional statistics approach for prediction of the wind direction. The interested reader is referred to some contributions in which several approaches have been proposed for forecasting wind direction. El-Fouly et al.^20^ suggested a linear time-series-based model for prediction of wind speed and direction. Garcia-Planas and Gongadze^21^ constructed a predictive model for wind speed and direction based on linear Markov chains under linear algebra point of view (see also Zeng et al.^22^, Fan et al.^23^, Zheng et al.^24^, Chen et al.^25^, Liu et al.^26^, Giangregorio et al.^27^, Wang et al.^28^). Note however that this paper approaches skew directional models from the Bayesian statistical angle.

Circular statistics can be applied to obtain the distribution of wind direction while Weibull, gamma, normal, Rayleigh, log-normal, inverse Gaussian, logistic distributions are some common models for the wind speed (see Deep et al.^9^ and Gugliani et al.^10^). For example, mixtures of von Mises (VM) distributions have been widely applied to model wind direction for different locations^10–17^. Gugliani et al.^18^ have applied Kato and Jones circular distribution^19^ to model wind direction.

However, wind datasets usually exhibit skew and multimodal patterns while most of the well-known circular distributions are symmetric such as the von Mises. Therefore in this paper, the application of skewed multimodal distributions is investigated for modeling the wind direction of South Africa hotspots from Bayesian viewpoint. The *k* sine-skewed von Mises (SSVM) distribution^29^ and mixtures of SSVM are ideal candidates to model wind direction data exhibiting both skewness and multimodality behaviour. Due to the fact that the likelihood-based inference and also the expectation maximization (EM) algorithm techniques for mixture models can be computationally complicated, a Bayesian approach can overcome such computational difficulties. It provides more accurate results for small datasets. Bayesian inference is conditional on the data and is exact, without reliance on asymptotic approximations. The Bayesian predictive posterior function can be used to forecast the wind direction.

Two important contributions of a Bayesian stochastic model are as follows:

(1) Inclusion of uncertainty about the parameters of the wind direction distribution results in using a more practical predictive distribution for the wind direction. This implies the predictive distribution is more disperse than the probability distributions when the uncertainty about the parameters is neglected. (2) The prior distributions of the parameters can represent the heterogeneity of the distributions of the wind direction over a wind farm. The wind direction distributions for various turbines on a farm may belong to the same family, such as the skew-von Mises, but the model parameters of each turbine may be different randomly according to some probability distributions. The Bayesian predictive distribution aggregates the non-homogeneous distributions into a single distribution that captures the variation among the probability distributions of the wind directions at the turbines’ locations on a wind farm.

There is a vast literature on the Bayesian approach for symmetric directional data specifically, Bayesian analysis using the symmetric von Mises and von Mises-Fisher distributions^30–39^. The von Mises-Fisher mixture model is implemented by Taghia et al.^40^ and Roge et al.^41^. Mulder et al.^42^ provided a Bayesian inference for mixtures of von Mises distributions using the reversible jump Markov chain Monte Carlo (MCMC) sampler and focused on noninformative priors. From the preceding it follows there is a gap in the literature that inspired us to propose novel Bayesian analysis of *skew directional wind data*. Recently Nakhaei Rad et al.^43,44^ provided Bayesian analysis for skew von Mises-Fisher distribution and skew Wrapped Cauchy mixture model.

In “Site location and wind data”, we provide details of the datasets that are analyzed in this paper. “Materials and methods” revisited the *k* sine-skewed von Mises distribution and the maximum likelihood estimates (MLEs) of the mixture of SSVM parameters. The Bayesian inference of the mixture of SSVM is also presented, followed by the posterior predictive distribution to forecast the wind direction. In “Evaluation and results”, a simulation study is conducted to show the performance of the proposed Bayesian approach. Finally, SSVM and mixture of SSVM are fitted to these datasets for different values of *k* together with their competitor, namely the mixture of von Mises distributions.

## Site location and wind data

**Figure 1:**
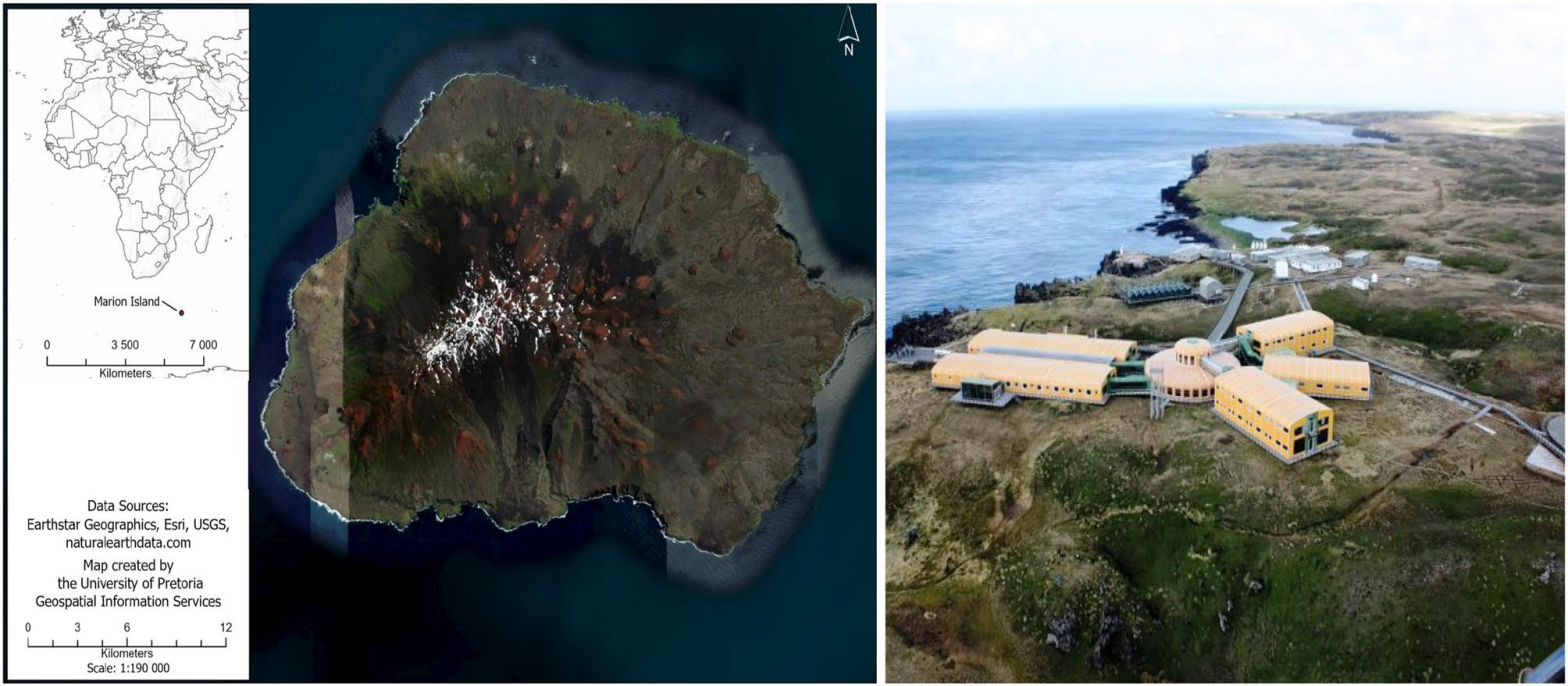
Marion island (created by the University of Pretoria) and meteorological research station on the island (provided by Antarctic Legacy of South Africa http://www.antarcticlegacy.org and https://blogs.sun.ac.za).

The first dataset (A) shows the wind direction of Marion island which is recorded daily at 08:00, 14:00 and 20:00 South Africa standard time (SAST) (relates to the main synoptic hours). Marion Island is part of South Africa with a climate that is highly oceanic in nature, coupled with the influence of passing frontal weather systems. In fact, the geographic location of Marion Island, lying directly in the path of eastward moving depressions all year round make it an excellent location for meteorological studies. Powerful regional winds, colloquially known as the ‘Roaring Forties’, so called as they have found between the latitudes of 40$^{\circ }$ and 50$^{\circ }$ in the Southern Hemisphere, blow almost every day in a north-westerly direction. The exceptional research potential of Marion Island for wind studies, as well the rate and impacts of climate change, is demonstrated by the presence of a permanent meteorological research station on the island. This station was established as early as 1948, and run by the South African National Antarctic Programme (see Fig. 1).

**Figure 2:**
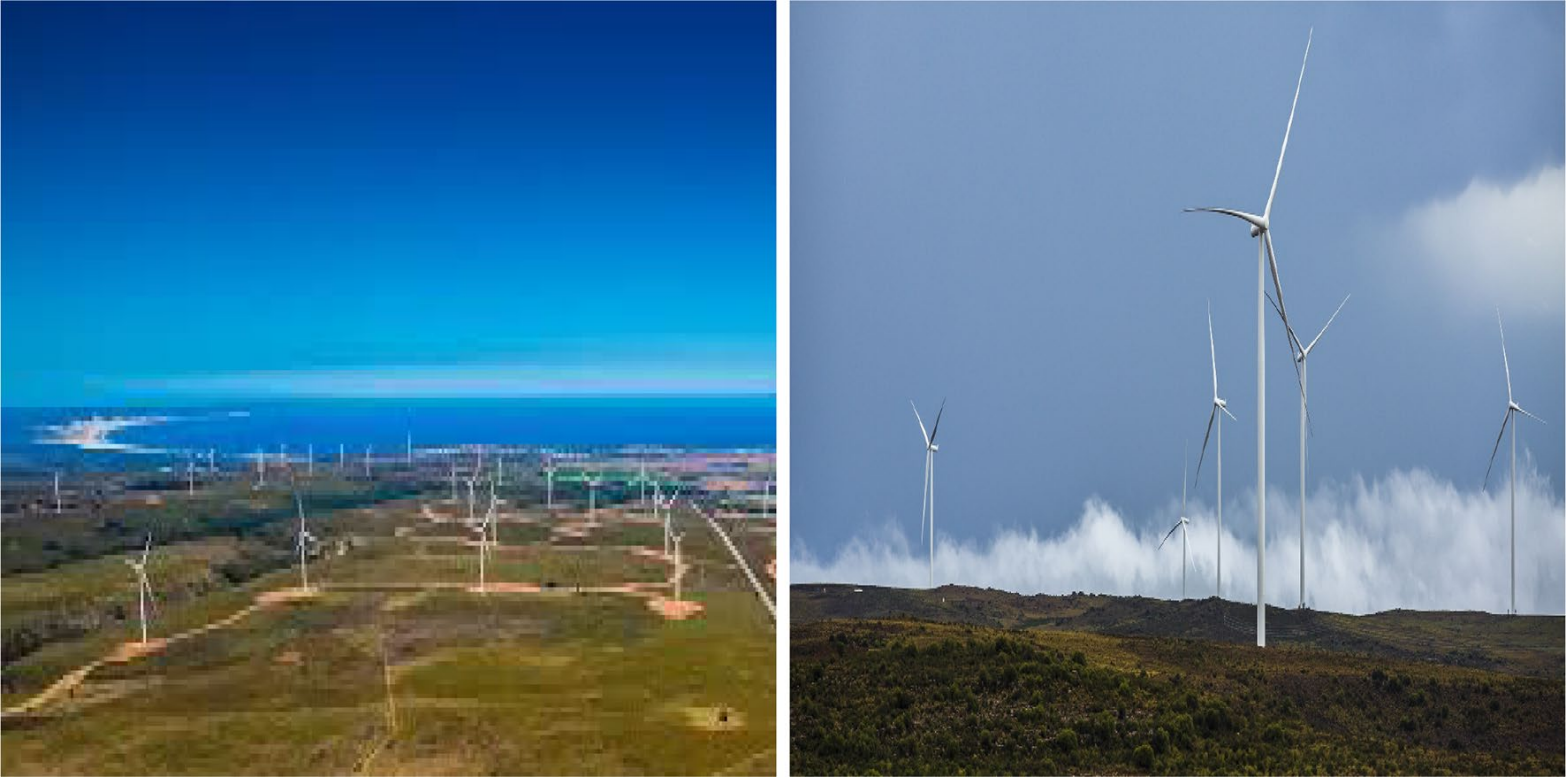
Jeffreys Bay (Humansdorp) wind farm https://jeffreysbaywindfarm.co.za (left) and Noupoort wind farm https://noupoortwind.co.za (right).

The second dataset (B) reflects the wind direction of Jeffreys Bay wind farm, recorded every 10 min at 60 m height. Jeffreys Bay is one of the biggest wind farms in South Africa spanning 3700 hectares with a 138 MW capacity. This site’s optimal wind conditions, relatively flat topography, minimal environmental constraints and its close proximity to the Eskom (electricity supply commission of South Africa) grid line, make it an ideal wind energy resource (see Fig. 2, left).

**Figure 3:**
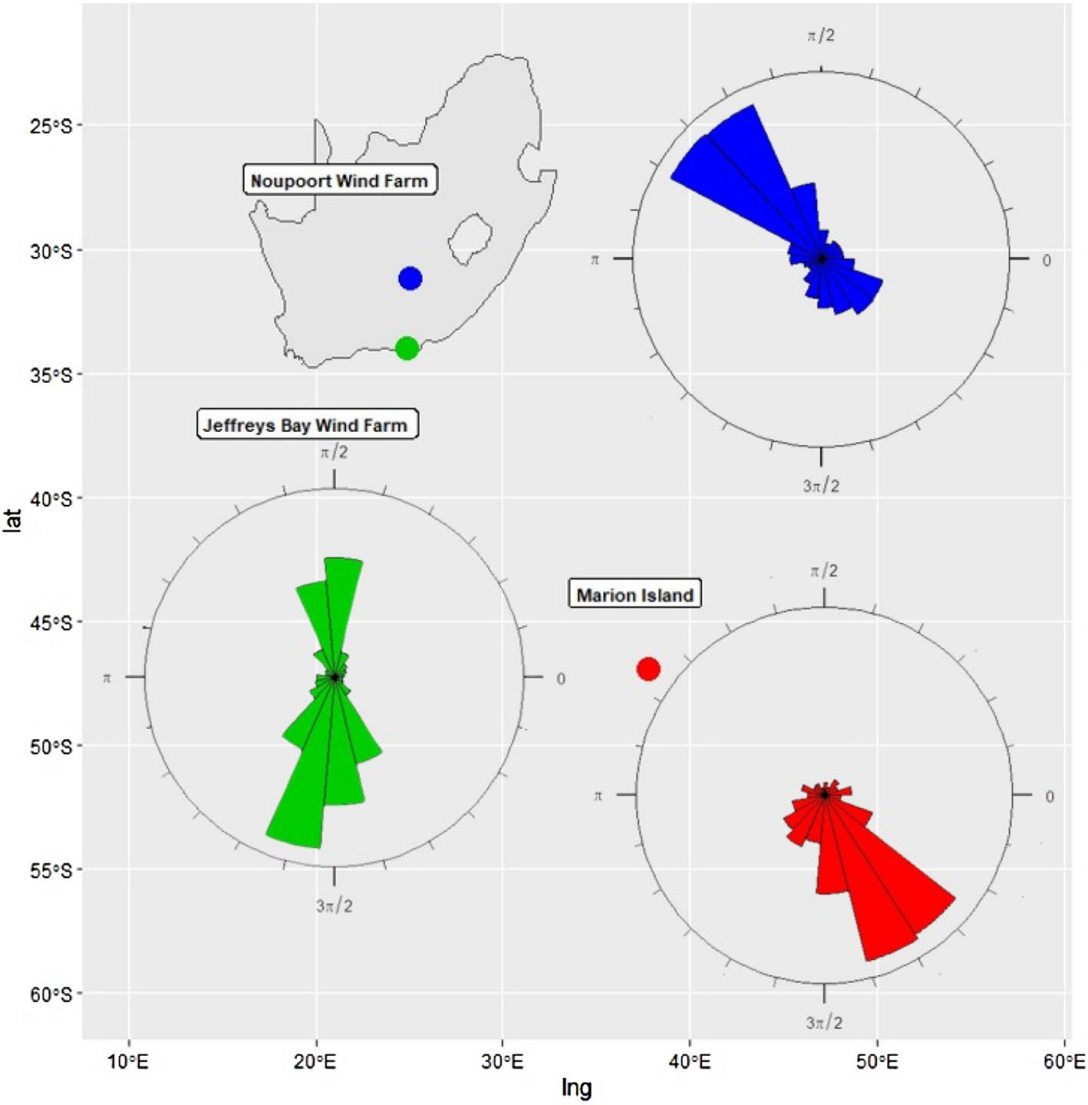
Map of South Africa with the locations of Marion island, Jeffreys Bay and Noupoort wind farms and rose plots of the wind direction (created by R programming language version 4.1.3 https://www.r-project.org).

The last dataset (C) shows wind direction of Noupoort wind farm comprising 7500 hectares and providing a 80 MW capacity, recorded every 10 min at 20 m height. This site is significant because of the excellent wind conditions, its proximity to national roads for wind turbine transportation, the favourable construction conditions, municipality and local stakeholder support and the straightforward electrical connection into the Eskom grid (see Fig. 2, right). Figure 3 shows the map of South Africa with the locations of Marion island, Jeffreys Bay and Noupoort wind farms and rose plots of the wind direction in these regions.

**Figure 4:**
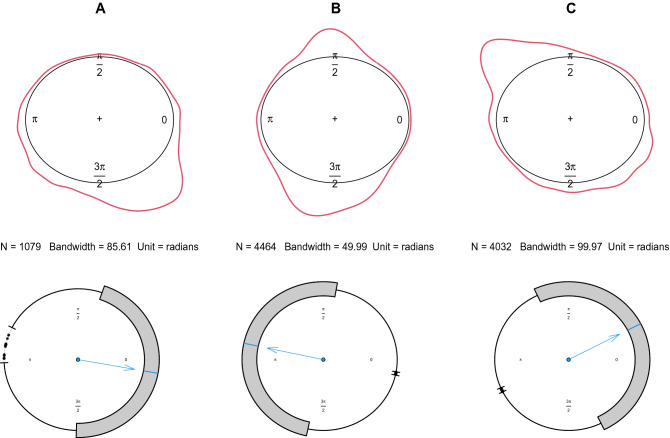
Boxplots and kernel density plots of the wind direction datasets A-C from Marion island, Jeffreys Bay and Noupoort wind farms.

Table 1 shows the descriptive information about the datasets. The results in Table 1, confirm skewness presence in these datasets. Also the Boxplots and kernel density plots of these datasets in Fig. 4. The Boxplots emphasize that these wind direction datasets reveal skew patterns and the kernel density plots confirm multimodal patterns. kernel density estimate is a smoothed version of the histogram which is a useful alternative to the histogram for continuous data. Unlike the histogram, the kernel technique produces a smooth estimate of the density function, uses all sample points’ locations and more convincingly suggests multimodality.

**Table 1:** Descriptive statistics for the wind direction data.

Id	Location	Begin	End	Duration (days)	n	Mean	Variance	Mean resultant length	Skewness	Kurtosis
A	Marion	01-Jan-2017	31-Dec-2017	365	1079	5.0242	0.4376	0.5624	0.4039	0.9686
B	Jeffreys Bay	01-Jan-2019	31-Jan-2019	31	4464	4.3498	0.7720	0.2279	0.5051	0.8084
C	Noupoort	01-Feb-2019	29-Feb-2019	29	4032	2.3351	0.7923	0.2076	− 0.1160	0.7220

## Materials and methods

### Sine-skewed von Mises distribution

**Figure 5:**
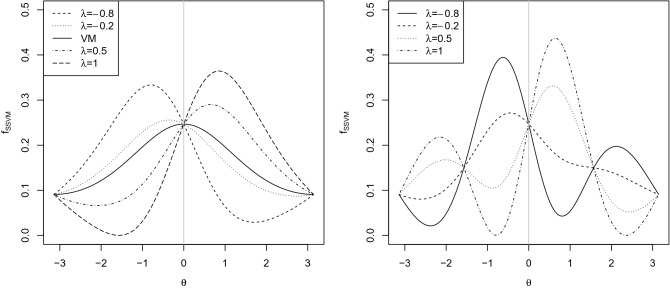
Density functions of the SSVM for $\tau =0.5$, $\mu =0$, $\lambda =-0.8,-0.2,0.5,1$ and $k=1$ (left) and $k=2$ (right).

Most of the distributions on the unit circle share the common feature of being symmetric about their location $\mu \in [-\pi ,\pi )$. However, since the assumption that data is symmetric is often rejected, Ref.^29^ introduced the *k* sine-skewed von Mises distribution with density function

1 $$\begin{aligned} f_{{SSVM}}(\theta ;\mu ,\tau ,\lambda )=\frac{1}{2\pi I_0(\tau )}\exp (\tau \cos (\theta -\mu ))(1+\lambda \sin (k(\theta -\mu ))), \end{aligned}$$

where $I_0(.)$ is the modified Bessel function of the first kind of order 0, $\mu \in [-\pi ,\pi )$ is the location parameter, $\tau >0$ is the concentration parameter, $-1\le \lambda \le 1$ is the skewness parameter and *k* is a positive integer. $\lambda >0$ leads to left skewed distributions and $\lambda <0$ provides right skewed distributions. The symmetric von Mises distribution is retrieved if $\lambda =0$. For $k\ge 2$, (1) has a multimodal form but for $k=1$ it can be both unimodal and bimodal. Figure 5 shows plots of SSVM density functions (see (1)) for $\mu =0$, $\tau =0.5$, $\lambda =-0.8,-0.2,0.5,1$ and $k=1,2$. As can be seen with $k=2$ bimodal distributions follows. A mixture of SSVM distributions with $M\in \mathbb {Z}^{+}$ components is expressed as

2 $$\begin{aligned} f_M(\theta ;{\varvec{w}},{\varvec{\mu }},{\varvec{\tau }},{\varvec{\lambda }})=\sum _{j=1}^{M} w_j f_{SSVM}(\theta ;\mu _j,\tau _j,\lambda _j), \end{aligned}$$

where ${\varvec{\mu }} = (\mu _1, \ldots ,\mu _M )$, ${\varvec{\tau }} = (\tau _1, \ldots ,\tau _M )$ and ${\varvec{\lambda }} = (\lambda _1, \ldots ,\lambda _M )$ are vectors of parameters, $\tau _j>0$, $\mu _j\in [-\pi ,\pi )$ and $\lambda _j\in [-1,1]$. ${\varvec{w}} = (w_1, \ldots ,w_M )$ is a vector of the weights containing the relative size of each component in the total sample satisfy the constraints $0 \le w_j \le 1$ and $\sum _{j=1}^{M} w_j=1$.

Algorithm 1^45^ can be used to generate a sample from the SSVM distribution in (1).
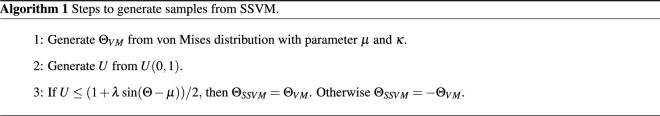


### Parameter estimation

In this section, first, the MLEs of parameters for a mixture of SSVM is presented, followed by a Bayesian inference when all the weight, location, concentration and skewness parameters $({\varvec{w}}$, ${\varvec{\mu }}$, ${\varvec{\tau }}$, ${\varvec{\lambda }})$ are unknown.

#### Maximum likelihood estimation

The log-likelihood function of a mixture of SSVM in (2), can be represented as follows:

3 $$\begin{aligned} l({\varvec{w}},{\varvec{\mu }},{\varvec{\tau }},{\varvec{\lambda }}|{\varvec{\theta }})= \sum _{i=1}^{n}log\left( \sum _{j=1}^{M} w_j f_{SSVM}(\theta _i;\mu _j,\tau _j,\lambda _j)\right) . \end{aligned}$$

By setting the partial derivatives of (3) with respect to (${\varvec{w}},{\varvec{\mu }},{\varvec{\tau }},{\varvec{\lambda }}$) to zero, the MLEs of $({\varvec{w}},{\varvec{\mu }},{\varvec{\tau }},{\varvec{\lambda }})$ can be obtained. Since no closed-form expressions exist, numerical methods should be used to obtain the estimates. The DEoptim package^46^ in R software which is based on the Differential Evolution (DE) algorithm^47^ is used to obtain the MLEs. Differential evolution is a heuristic evolutionary method for global optimization that is effective in many problems of interest in science and technology and its significant performance as a global optimization algorithm on continuous numerical minimization problems has been extensively studied^48^. DEoptim has made this algorithm possible to easily apply in the R language and environment. DEoptim relies on repeated evaluation of the objective function in order to move the population toward a global minimum^46^.

#### Bayes estimation

Let ${\varvec{\theta }}=(\theta _1,\theta _2,\ldots ,\theta _n)$ be a random sample of size *n* from a mixture of SSVM (see (2)). It should be noted that the number of components *M* is considered as a known parameter. Suppose the latent variable ${\varvec{d}}=(d_1,\ldots ,d_n)$ allocates the component that ${\varvec{\theta }}$ is sampled from. The probability of being attributed to component *j* is given by

$$\begin{aligned} P(d_i = j|{\varvec{w}}) = w_j. \end{aligned}$$

Therefore, for $i = 1, \ldots , n$ and $j = 1, \ldots , M$

$$\begin{aligned} f(\theta _i|d_i = j) = f_{SSVM}(\theta _i;\mu _j, \tau _j,\lambda _j). \end{aligned}$$

It implies that conditional on $d_i$, $\theta _i$ is an independent observation from its respective component *j* that makes the inference easier because the problem reduces to inference for a single SSVM component. Therefore, conditional on ${\varvec{d}}$, the likelihood function can be expressed as

4 $$\begin{aligned} L({\varvec{\mu }},{\varvec{\tau }},{\varvec{\lambda }}|{\varvec{\theta }},{\varvec{d}})= \prod _{i=1}^{n} f_{SSVM}(\theta _i;\mu _{d_i},\tau _{d_i},\lambda _{d_i}). \end{aligned}$$

Subsequently, we measure the uncertainty in the parameters with the following prior distributions for $({\varvec{w}},{\varvec{\mu }},{\varvec{\tau }},{\varvec{\lambda }})$. If the sample size is small, or available data provides only indirect information about the parameters of interest, the prior distribution becomes more important^49^. Ghaderinezhad et al.^50^ implemented the Wasserstein impact measure (WIM) as a measure of quantifying prior impact. It helps us to choose between two or more given priors. Nakhaei Rad et al.^44^ by using the WIM measure demonstrated that the combination of the von Mises, gamma and truncated normal distributions decreases the execution time in the Gibbs sampling algorithm. Thus, providing accurate parameter estimates for the skew Fisher-von Mises distribution^51^ as well.

Therefore, consider independent von Mises and gamma distributions with parameters $({\varvec{\mu }_0},{\varvec{\tau }_0})$ and $({\varvec{\alpha }},{\varvec{\beta }})$ as priors for ${\varvec{\mu }}$ and ${\varvec{\tau }}$, respectively:

5 $$\begin{aligned} \pi (\mu _j,\tau _j;{\mu _0}_{j},{\tau _0}_{j},\alpha _j,\beta _j)\propto \exp ({\tau _0}_{j}\cos (\mu _j-{\mu _0}_j))\tau _{j}^{\alpha _j-1}\exp (-\beta _j\tau _j), \end{aligned}$$

where $${\tau _0}_j,\alpha _j,\beta _j>0$$, $${\mu _0}_j\in [-\pi ,\pi )$$ and $$j=1,2,\ldots ,M$$.

For the skewness parameter ${\varvec{\lambda }}$, the truncated normal distribution on $[-1,1]$ is proposed with parameters ${\varvec{\xi }}$ and ${\varvec{\sigma }}^2$:

6 $$\begin{aligned} \pi (\lambda _j; \xi _j,\sigma _j)=\frac{1}{\sigma _j}\frac{\phi \left( \frac{\lambda _j-\xi _j}{\sigma _j}\right) }{\Phi \left( \frac{1-\xi _j}{\sigma _j}\right) -\Phi \left( \frac{-1-\xi _j}{\sigma _j}\right) },~~~~~~~~\lambda _j\in [-1,1]. \end{aligned}$$

where $\xi _j\in \mathbb {R}$, $\sigma _j>0$, $j=1,2,\ldots ,M$, $\phi (.)$ is the density function of standard normal distribution and $\Phi (.)$ is its cumulative distribution function.

For the weight parameter ${\varvec{w}}$, the Dirichlet distribution with parameter ${\varvec{c}}$ is considered as prior:

7 $$\begin{aligned} \pi ({\varvec{w}};{\varvec{c}})= \frac{1}{B({\varvec{c}})}\prod _{j=1}^{M}w_j^{c_j-1}, \end{aligned}$$

where $c_j>0$ for $j=1,\ldots ,M$ and $B({\varvec{c}})=\frac{\prod _{j=1}^{M}\Gamma (c_j)}{\Gamma \left( \sum _{j=1}^{M}c_j\right) }$. Thus the marginal distribution of $w_j$ is $Beta(c_j,\sum _{i=1}^{M}c_i-c_j)$^52^.

Subsequently, the posterior distribution is:

8 $$\begin{aligned} \pi ({\varvec{w}},{\varvec{\mu }},{\varvec{\tau }},{\varvec{\lambda }}|{\varvec{\theta }})\propto \pi ({\varvec{w}},{\varvec{\mu }},{\varvec{\tau }},{\varvec{\lambda }})L({\varvec{w}},{\varvec{\mu }},{\varvec{\tau }},{\varvec{\lambda }}|{\varvec{\theta }}), \end{aligned}$$

with $\pi ({\varvec{w}},{\varvec{\mu }},{\varvec{\tau }},{\varvec{\lambda }})$ from (5), (6) and (7). The full conditionals of parameters $({\varvec{w}},{\varvec{\mu }},{\varvec{\tau }},{\varvec{\lambda }},{\varvec{d}})$ for using in the Gibbs algorithm follow from (8). Therefore the Gibbs sampler is as follows (see Algorithm 2):
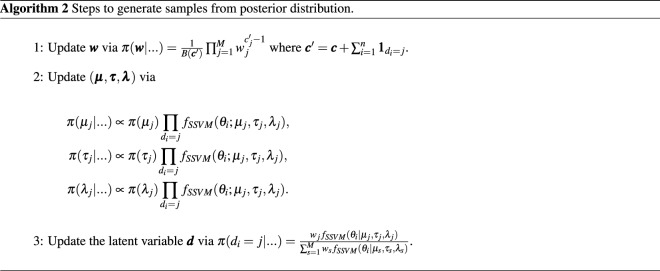


For ${\varvec{\theta }}=(\theta _1,\theta _2,\ldots ,\theta _n)$, a set of observations and ${\varvec{\varpi }}=({\varvec{w}},{\varvec{\mu }},{\varvec{\tau }},{\varvec{\lambda }})$, the posterior predictive distribution for a new data point $\theta _{new}$ and $d_{new}$ (the corresponding latent switch variable associated with $\theta _{new}$) is:

$$\begin{aligned} \pi (\theta _{new}|{\varvec{\theta }})=\sum _{d_{new}}\int _{{\varvec{\varpi }}}f(\theta _{new}| d_{new},{\varvec{\mu }},{\varvec{\tau }},{\varvec{\lambda }}) p(d_{new}| {\varvec{w}})\pi ({\varvec{\varpi }}|{\varvec{\theta }})d{\varvec{\varpi }}, \end{aligned}$$

where $\theta _{new}$ is independent of the sample data ${\varvec{\theta }}$. Sometimes the form of $\pi (\theta _{new}|{\varvec{\theta }})$ can be derived directly, but it is often easier to sample from $\pi (\theta _{new}|{\varvec{\theta }})$ using Monte Carlo methods. For generating an iid sample $(\theta ^{(1)}_{new},\theta ^{(2)}_{new},\ldots ,\theta ^{(n)}_{new})$ from $\pi (\theta _{new}|{\varvec{\theta }})$ Algorithm 3 is followed:
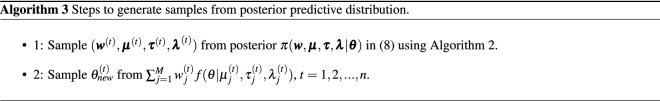


### Model selection criteria

Model selection is an important part of any statistical analysis and many tools for selecting the “best model” have been suggested in the literature. Here, three different criteria are applied to evaluate the models. Suppose ${\varvec{\varpi }}$ is the vector of parameters with *k* elements, $l({\varvec{\varpi }}| {\varvec{\theta }})$ is the log-likelihood function and *n* is the sample size. The Akaike information criterion (AIC)^53^ and the Bayesian information criterion (BIC)^54^ as penalized-likelihood criteria are given by

$$\begin{aligned} AIC&=-2l({\varvec{\varpi }}|{\varvec{\theta }})+2k,\\ BIC&=-2l({\varvec{\varpi }}|{\varvec{\theta }})+k\log n. \end{aligned}$$

As can be seen, BIC penalizes parameters more heavily than AIC. Spiegelhalter et al.^55^ proposed the deviance information criterion (DIC), as

$$\begin{aligned} DIC=2\overline{D}({\varvec{\varpi }})-D(\overline{{\varvec{\varpi }}}), \end{aligned}$$

where $D({\varvec{\varpi }})=-2 l({\varvec{\varpi }}|{\varvec{\theta }})$, $\overline{{\varvec{\varpi }}}$ is the posterior mean of ${\varvec{\varpi }}$ and $\overline{D}(.)$ is the average of *D*(.) over the samples of ${\varvec{\varpi }}$. DIC is usually applied in Bayesian model selection problems where the posterior distribution has been obtained by MCMC simulation.

## Evaluation and results

### Simulation

**Figure 6:**
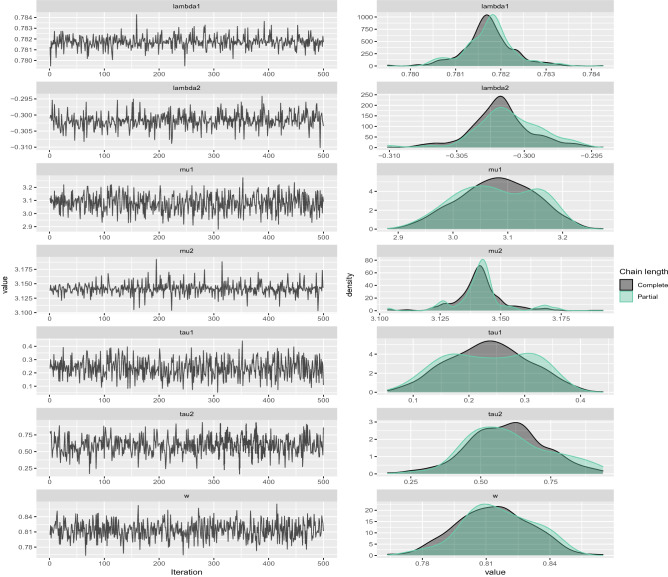
Traceplots and estimated posterior density plots of generated samples for $(w,\mu _1,\tau _1,\lambda _1,\mu _2,\tau _2,\lambda _2)$ in Table 3 for $n=500$.

In this section, to assess the performance of the proposed Bayesian approach a simulation study was conducted to estimate the parameters of SSVM in (1) and mixture of SSVM in (2). SSVM with parameters $\mu =3,\tau =2,\lambda =0.5$ and prior parameters $\mu _0=0,\tau _0=0.01,\alpha =4,\beta =2,\xi =0.5$, $\sigma =0.01$ and a mixture of SSVM with two components ($M=2$) with parameters $w=0.8, \mu _1=3, \tau _1=0.2, \lambda _1=0.75, \mu _2=3.14, \tau _2=0.6, \lambda _2=-0.3$ and prior parameters $\mu _{0_1}=3$, $\tau _{0_1}=0.1$, $\alpha _1=4$, $\beta _1=2$, $\xi _1=0.9$, $\sigma _1=0.15$, $c_1=1$ and $\mu _{0_2}=0$, $\tau _{0_2}=0.1$, $\alpha _2=6$, $\beta _2=2$, $\xi _2=-1$, $\sigma _2=1.0$, $c_2=1$ were considered. Samples of sizes $n=50,100,500$ were generated from the posterior distribution in (8) for each model, using Gibbs sampling in Algorithm 2. The Bayes estimates of parameters were obtained based on the squared error and absolute error loss functions. The posterior mean and the posterior median are the Bayes estimators under the squared error and absolute error loss functions, respectively. In order to obtain the Bayes estimates of the parameters, the mean and median of the generated samples from the posterior distribution (8) were calculated along with some other descriptive statistics. The results, including the sample mean, standard deviation (sd) and quartiles (Q1, median, and Q3) of the posterior distribution are summarized in Tables 2 and 3. As can be seen the differences between true values of the parameters and the posterior sample mean and the posterior sample median are minimal. Therefore, the proposed Bayesian approach provides accurate estimates for the parameters. The traceplots of the generated samples from the posteriors and the compare-partial plots^56^ are shown in Fig. 6 for the mixture of SSVM. A traceplot is used for evaluating convergence which shows the time series of the sampling process from the posterior distribution. It is expected to get a traceplot that looks completely random. A compare-partial plot provides overlapped kernel density plots related to the last part of the chain (the last 10 values, in green) and the whole chain (in black). The overlapped kernel densities are expected to be similar. It means the initial and final parts of the chain should to be sampling in the same target posterior distribution. These plots in Fig. 6 confirm the convergence of the chains and show that the Gibbs sampler recovers the values that actually generate the dataset.

**Table 2:** Bayes estimates of parameters of SSVM with prior parameters, $\mu _0=0,\tau _0=0.01,\alpha =4,\beta =2,\xi =0.5$ and $\sigma =0.01$.

	Parameter	Actual value	Mean	SD	$Q_1$	Median	$Q_3$
$n=500$	$\mu$	3.00	2.9634	0.2267	2.7158	2.9990	3.2771
	$\tau$	2.00	1.9826	0.4958	1.1513	1.9568	3.0635
	$\lambda$	0.50	0.4915	0.0084	0.4858	0.4919	0.5012
$n=100$	$\mu$	3.00	3.1094	0.0443	2.9982	3.1180	3.1753
	$\tau$	2.00	2.0177	0.3556	1.3373	2.0351	2.6634
	$\lambda$	0.50	0.4836	0.0240	0.4592	0.4712	0.5342
$n=50$	$\mu$	3.00	3.1925	0.0380	3.0926	3.1954	3.2491
	$\tau$	2.00	1.9310	0.2712	1.3405	1.9381	2.4485
	$\lambda$	0.50	0.5214	0.0220	0.4669	0.5306	0.5390

**Table 3:** Bayes estimates of parameters of a mixture of SSVM with prior parameters, $\mu _{0_1}=3$, $\tau _{0_1}=0.1$, $\alpha _1=4$, $\beta _1=2$, $\xi _1=0.9$, $\sigma _1=0.15$, $c_1=1$ and $\mu _{0_2}=0$, $\tau _{0_2}=0.1$, $\alpha _2=6$, $\beta _2=2$, $\xi _2=-1$, $\sigma _2=1.0$, $c_2=1$.

	Parameter	Actual value	Mean	SD	$Q_1$	Median	$Q_3$
$n=500$	*w*	0.80	0.8135	0.0172	0.7820	0.8132	0.8453
	$\mu _1$	3.00	3.0803	0.0685	2.9469	3.0810	3.2094
	$\tau _1$	0.20	0.2357	0.0710	0.0996	0.2352	0.3673
	$\lambda _1$	0.75	0.7817	0.0005	0.7806	0.7817	0.7829
	$\mu _2$	3.14	3.1413	0.0100	3.1223	3.1412	3.1654
	$\tau _2$	0.60	0.5925	0.1354	0.3143	0.5969	0.8621
	$\lambda _2$	$-0.30$	$-0.3017$	0.0022	$-0.3067$	$-0.3017$	$-0.2965$
$n=100$	*w*	0.80	0.8419	0.0334	0.7775	0.8395	0.9014
	$\mu _1$	3.00	3.1114	0.0516	3.0224	3.1101	3.2243
	$\tau _1$	0.20	0.1945	0.0554	0.0977	0.1871	0.3186
	$\lambda _1$	0.75	0.7316	0.0028	0.7269	0.7314	0.7373
	$\mu _2$	3.14	3.1413	0.0058	3.1322	3.1427	3.1579
	$\tau _2$	0.60	0.5964	0.1206	0.3761	0.5998	0.8152
	$\lambda _2$	$-0.30$	$-0.3326$	0.0038	$-0.3410$	$-0.3320$	$-0.3266$
$n=50$	*w*	0.80	0.8351	0.0487	0.7383	0.8360	0.9212
	$\mu _1$	3.00	3.2101	0.0789	3.1226	3.2164	3.3998
	$\tau _1$	0.20	0.1903	0.0665	0.0847	0.1912	0.3147
	$\lambda _1$	0.75	0.7320	0.0032	0.7270	0.7314	0.7378
	$\mu _2$	3.14	3.1420	0.0033	3.1342	3.1418	3.1489
	$\tau _2$	0.60	0.6164	0.1145	0.3946	0.6158	0.7955
	$\lambda _2$	$-0.30$	$-0.3321$	0.0033	$-0.3390$	$-0.3321$	$-0.3274$

**Figure 7:**
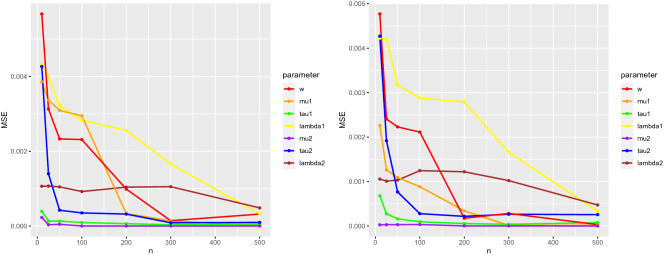
MSE of Bayes estimates under the squared error (left) and absolute error (right) loss functions, for $n = 10,25,50,100,200,300,500$.

To evaluate the accuracy of the obtained Bayes estimates, the mean squared errors (MSE) of the estimates under squared error and absolute error loss functions for the mixture of SSVM with two components ($M=2$) with parameters which are mentioned above were obtained for different sample sizes $n = 10,25,50,100,200,300,500$ with 100 repetitions. The results in Fig. 7 show that by increasing *n*, MSE decreases and also, the MSEs of the estimates for absolute error loss function are less than squared error loss function because outliers have a smaller effect on the median.

### Real data

To demonstrate the performance of the SSVM for the wind direction data for South African hotspots, three real skewed datasets as discussed in “Site location and wind data” (see Table 1) were analyzed. Due to the multimodal pattern of the datasets observed in Fig. 4, the following distributions were assumed:

- mixtures of von Mises distributions with $M=2,3,4$ components,
- SSVM with $$k=2$$,
- mixtures of SSVM with $k=1$ and $M=2$ components,
- mixtures of SSVM with $k=2$ and $M=2$ components.

**Figure 8:**
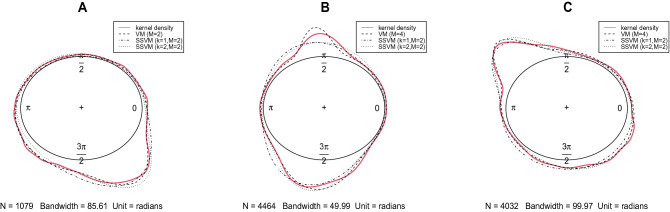
Kernel density plots of datasets and fitted curves based on MLEs.

The MLEs of parameters $(\mu ,\tau ,\lambda ,p)$ were obtained by using the DEoptim package in R. The results including MLEs and corresponding log-likelihood, AIC and BIC are reported in Table 4. A model with the maximum log-likelihood and minimum values of AIC and BIC provides better fit for the data. Therefore, for dataset A, the mixture of SSVM with $k=1$ provides the best fit. Mixture of SSVM with $k=2$ and the mixture of von Mises with $M=2$ are the second and third best models, respectively. For datasets B and C, the mixture of SSVM with $k=2$ provides the best fit and the mixture of von Mises with $M=4$ is the second best model. In all of these datasets, the difference in the AIC and BIC values of the mixture of SSVM in comparison to the mixture of von Mises are remarkable. Furthermore, the mixture of SSVM with smaller value of *M*, outperformed the mixture of von Mises. The kernel density plots of the datasets and the fitted curves consisting of the best mixture of von Mises and mixture of SSVM for $k=1,2$ are shown in Fig. 8.

**Table 4:** Maximum likelihood estimates and corresponding log-likelihood, AIC and BIC for datasets.

Data	Model	$\hat{\tau }$	$\hat{\mu }$	$\hat{\lambda }$	$\hat{w}$	Log-likelihood	AIC	BIC
**A**	Mixture of VM ($M=2$)	0.8264	4.6437	–	0.6388	$-1524.7750$	3059.5490	3084.4680
		13.4279	5.2866	–	0.3612			
	Mixture of VM ($M=3$)	1.6861	4.0380	–	0.2852	$-2241.4770$	4498.9530	4538.8230
		11.7538	2.2718	–	0.4200			
		0.6421	5.6606	–	0.2948			
	Mixture of VM ($M=4$)	1.6430	4.0014	–	0.3179	$-1522.9620$	3067.9240	3122.7460
		9.2575	5.1609	–	0.1863			
		0.7727	6.0288	–	0.2365			
		13.9738	5.3294	–	0.2591			
	**Mixture of SSVM** (${\varvec{k}=1,\varvec{M}=2}$)	0.5490	3.4434	0.8831	0.4291	$\mathbf{-1248.6560}$	**2511.3130**	**2546.2000**
		5.9863	5.2451	0.0447	0.5709			
	SSVM ($k=2$)	1.3283	4.8196	0.4113	–	$-1575.1960$	3156.3930	3171.3440
	Mixture of SSVM ($k=2,M=2$)	0.7644	4.4362	0.5208	0.5974	$-1437.3610$	2888.7220	2923.6090
		11.8642	5.2842	0.1428	0.4026			
**B**	Mixture of VM ($M=2$)	3.9011	4.5829	–	0.6284	$-6392.3200$	12794.6400	12826.6600
		4.1262	1.6053	–	0.3716			
	Mixture of VM ($M=3$)	0.6536	1.8472	–	0.2602	$-6066.5200$	12149.0400	12200.2700
		6.8578	4.6102	–	0.5356			
		37.5722	1.6121	–	0.2042			
	Mixture of VM ($M=4$)	1.2487	1.5608	–	0.2000	$-6060.9610$	12143.9200	12214.3600
		39.7624	1.6653	–	0.1962			
		1.5231	4.1872	–	0.1187			
		7.5915	4.6293	–	0.4851			
	Mixture of SSVM ($k=1,M=2$)	3.7053	1.6860	0.4816	0.3799	$-6295.287$	12604.5700	12649.4000
		4.2219	4.5949	− 0.7337	0.6201			
	GSSVM ($k=2$)	0.4141	3.8738	0.6329	–	$-6441.0300$	12888.0600	12907.2700
	**Mixture of SSVM** (${\varvec{k}=2,\varvec{M}=2}$)	1.2525	2.1711	− 0.8901	0.4731	$\mathbf{-5372.1610}$	**10758.3200**	**10803.1500**
		7.3277	4.6315	− 0.2355	0.5269			
**C**	Mixture of VM ($M=2$)	0.9550	5.3272	–	0.5384	$-6238.2750$	12486.5500	12518.0600
		10.1064	2.2563	–	0.4616			
	Mixture of VM ($M=3$)	2.4565	5.3344	–	0.2757	$-6203.316$	12422.6300	12473.0500
		0.1095	2.3723	–	0.3075			
		12.3062	2.2591	–	0.4168			
	Mixture of VM ($M=4$)	1.8131	5.3286	–	0.4136	$-6187.3030$	12396.6100	12465.9300
		1.3339	2.2543	–	0.1532			
		24.8131	2.2987	–	0.2757			
		3.0057	2.1467	–	0.1573			
	Mixture of SSVM ($k=1,M=2$)	0.8520	5.0994	− 0.2553	0.5582	$-6220.1690$	12454.3400	12498.4500
		10.9951	2.2543	0.7743	0.4418			
	SSVM ($k=2$)	0.3378	2.9753	− 0.7547	–	$-6529.6970$	13065.3900	13084.3000
	**Mixture of SSVM** (${\varvec{k}=2,\varvec{M}=2}$)	0.4357	4.6249	0.7508	0.6137	$\mathbf{-5584.4470}$	**11182.8900**	**1127.0100**
		14.8628	2.2538	0.0835	0.3863			

The best model is indicated in bold.

**Figure 9:**
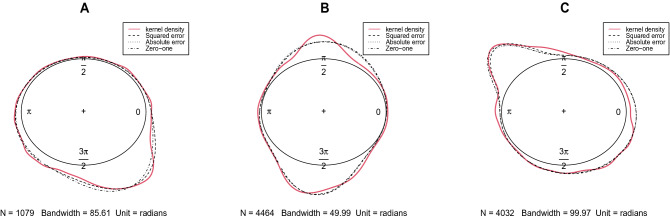
Kernel density plots of datasets and fitted curves based on Bayes estimates.

To demonstrate the performance of the proposed Bayesian approach, a mixture of two SSVM distributions is fitted to dataset A for $k=1$, and to dataset B and C with $k=2$. A sample of size $n=500$ was generated from the posterior distribution in (8) for each model, using the Gibbs sampling outlined in Algorithm 2. The Bayes estimates of the parameters were obtained based on the squared error, absolute error and zero-one loss functions. For our purpose, the posterior mean, posterior median and posterior mode were calculated from the generated samples as the Bayes estimates of parameters under the different mentioned loss functions. The results including the Bayes estimates of the parameters and corresponding DIC are reported in Table 5. A model with minimum value of DIC has better fit for the data. The mentioned models above with parameters estimated based on the absolute error loss function provide more accurate fit for the datasets. The kernel density plots of the datasets and the fitted curves are shown in Fig. 9.

**Table 5:** Bayes estimates of parameters under different loss functions and corresponding DIC for datasets.

Data	Model	Loss function	$\hat{\tau _1}$	$\hat{\mu _1}$	$\hat{\lambda _1}$	$\hat{\tau _2}$	$\hat{\mu _2}$	$\hat{\lambda _2}$	$\hat{w}$	DIC
A	Mixture of SSVM (${k=1,M=2}$)	Squared error	0.4609	3.4528	0.7013	6.1235	5.2314	0.3395	0.5002	3086.42
		Absolute error	0.4368	3.4715	0.6973	6.0937	5.2244	0.3398	0.4997	**3086.15**
		Zero-one	0.3274	3.4560	0.5831	5.8139	5.1360	0.3334	0.4881	3087.48
B	Mixture of SSVM (${k=2,M=2}$)	Squared error	1.5323	2.0121	− 0.8772	7.4175	4.6898	− 0.2405	0.4997	12839.28
		Absolute error	1.5743	2.0463	− 0.8969	7.3480	4.6784	− 0.2303	0.4996	**12837.10**
		Zero-one	1.5048	1.9889	− 0.9049	7.3433	4.6634	− 0.1981	0.5046	12885.87
C	Mixture of SSVM (${k=2,M=2}$)	Squared error	0.4832	4.6294	0.7985	14.8946	2.2955	0.0995	0.6087	12796.50
		Absolute error	0.4038	4.6262	0.7794	14.6273	2.3348	0.0898	0.6122	**12795.83**
		Zero-one	0.4014	4.6227	0.7811	15.5572	2.3375	0.0829	0.6044	12812.50

The best model is indicated in bold.

In Table 6, using Algorithm 3, the predicted means of wind direction were obtained, based on absolute error loss function, for $n=20,50,100$. Also, $95\%$ credible intervals are derived. We focused on the assumption of absolute error loss function as a result of the performance observed in Table 5. As can be seen, by increasing *n*, the mean value of the predictive wind direction distributions are getting closer to the mean value of the datasets. In addition, the length of the credible intervals is short. Therefore, our approach provides accurate prediction of wind direction.

**Table 6:** Predicted wind direction based on absolute error loss function for different values of *n*.

Data	Mean	Model	*n*	Predicted mean	$95\%$ Credible interval
A	5.0242	SSVM (${{k}=1,{M}=2}$)	20	4.8754	4.4275,5.3233)
			50	5.1249	4.4611,5.3887)
			100	5.0171	(4.7442,5.2900)
B	4.3498	SSVM (${{k}=2,{M}=2}$)	20	4.4918	(3.6417,5.3419)
			50	4.4652	(3.9834,4.9470)
			100	4.3580	(3.8963,4.8198)
C	2.3351	SSVM (${{k}=2,{M}=2}$)	20	2.5216	(1.7277,3.3154)
			50	2.2784	(1.7142,2.8426)
			100	2.3726	(1.9737,2.7714)

## Conclusion

In this paper, due to the skew and multimodal patterns of wind direction datasets from South Africa, a skew and multimodal mixture model, namely mixture of sine-skewed von Mises distributions is proposed for modeling wind direction. Our proposed model outperforms mixtures of von Mises distributions (with larger number of components) which is extensively used in literature to model wind direction. Due to the difficulties in estimating parameters for mixture models using maximum likelihood method, a Bayesian approach is implemented for estimating the parameters of a mixture of sine-skewed von Mises distributions using a Gibbs sampler. The results show this approach provides accurate estimates for parameters. In addition the posterior predictive distribution can be applied for wind direction prediction (see Table 6) which provides accurate forecasts. Future work may consist of implementing the models of Bekker et al.^57^ and Kato and Jones^19^ and investigating the impact of other prior choices^50^. One can use our proposal to improve the wind energy potential as described and detailed in Arashi et al.^58^.

## Data Availability

The datasets used and/or analysed during the current study available from the corresponding author on reasonable request.
